# Invasive lobular breast carcinoma variants; clinicopathological features and patient outcomes

**DOI:** 10.1007/s10549-025-07729-z

**Published:** 2025-05-21

**Authors:** Aysegul Aktas, Meryem Gunay Gurleyik, Dogukan Akkus, Zekeriya Ucur, Fugen Aker

**Affiliations:** 1 General Surgery, Haydarpasa Numune Training and Research Hospital, University of Health Sciences Turkiye, SBU Haydarpasa Numune Egitim Ve Arastırma Hastanesi, Selimiye, Tibbiye Caddesi, No:23, 34668 Uskudar Istanbul, Turkey; 2 Pathology,, Haydarpasa Numune Training and Research Hospital, University of Health Sciences Turkiye, Istanbul, Turkey

**Keywords:** Breast cancer, Classic variant, Invasive lobular breast carcinoma, Pleomorphic lobular carcinoma, Surgery, Survival

## Abstract

**Introduction:**

An understanding of the differences among the invasive lobular breast carcinoma (ILC) variants is crucial for risk stratification, and tailored treatment planning. This article compares variants of ILC according to their clinical outcomes and histopathological features.

**Patients and methods:**

Patients diagnosed with ILC between January 2010 and August 2021 were retrospectively evaluated. Patients were divided into three groups; 1: classic ILC (cILC); 2: pleomorphic lobular carcinoma (PLC); 3: mixed ILC. Mixed ILC was divided into three subgroups: 3a, cILC + PLC; 3b, cILC + mixed; 3c, PLC + mixed.

**Results:**

A total of 254 patients were included in the study. Median overall survival (OS) was 48 months, and median disease-free survival (DFS) was 46 months. Locoregional recurrence (LRR) occurred in 15 (5.9%) of the patients, and distant metastasis (DM) developed in 23 (9.1%). Death occurred in 16 (6.3%) patients. There was no significant difference in LRR rate among groups. When considering five groups (Groups 1, 2, 3a, 3b, and 3c), the median OS was 62.5, 52.0, 50.8, 56.7, and 41.5 months, respectively, while the median DFS was 60.3, 46.6, 46.7, 54.5, and 39.6 months, respectively. Notably, the PLC + mixed group without a classic variant (Group 3c) exhibited even worse outcomes than pure PLC.

**Conclusions:**

In this study, pure cILC exhibited the best prognostic features among the ILC variants. Furthermore, we observed a higher mastectomy rate in patients with pleomorphic variants. Surgical management of ILC remains controversial. Moreover, comprehensive randomized controlled trials are essential to establish standardized treatment protocols for ILC patients.

## Introduction

Breast cancer continues to be a significant concern among women worldwide. Of the several diverse subtypes, invasive lobular carcinoma (ILC) is the second-most common histopathological subtype, accounting for 10–15% of all breast cancers [1–3]. The incidence of ILC has increased in recent years in association with hormone replacement therapy [2, 4, 5]. ILC is more common in older women and tends to be multifocal/multicentric. Bilateral breast involvement is more common in ILC patients [6, 7]. Loss of the adhesion protein E-cadherin causes a linear “discohesive” growth pattern. Therefore, ILC tends to grow diffusely, often without a significant desmoplastic reaction, and is often difficult to distinguish from surrounding breast tissue on both mammography and ultrasonography, leading to detection at a more advanced stage [8, 9]. ILC of the breast represents a heterogeneous group of tumors that can be classified into subgroups based on their architectural growth patterns and cytomorphological features. The 5th edition of the World Health Organization (WHO) classification includes three architectural variants (alveolar, solid, and tubulolobular ILC) and three cytological variants (histiocytoid, signet-ring cell, and pleomorphic ILC). In addition, other ILC variants have been identified, such as ILC with extracellular mucin pools (mucinous) and solid papillary ILC [7, 10]. Each variant has different characteristics. cILC is the most common variant, characterized by single-file rows of tumor cells with low to intermediate nuclear grade. The alveolar variant demonstrates a more cohesive growth pattern than cILC. The solid variant often shows a prominent inflammatory response with little or no intervening stroma, and is associated with higher grades, pleomorphism, mitosis, and Ki-67 proliferation index than cILC, in addition to an association with worse outcomes. The signet-ring cell variant may mimic gastric cancer if metastasis occurs. The histiocytoid variant usually shows a high nuclear grade and is mostly of the Luminal B molecular subtype; nevertheless, it is associated with a better prognosis and is rarely seen [7, 10, 11]. ILC variants seldom present in pure form; they are more likely to be associated with the classic type. Furthermore, there is no specific cut-off for defining ILC variants, and some may not be recognized as distinct subtypes [7, 12–15]

Low-grade estrogen receptor (ER)-positive ILC is generally associated with a good prognosis, but some studies have demonstrated that the long-term outcomes of ILC are inferior to those of invasive ductal carcinoma (IDC) of the same stage [16]. Classic ILC (cILC) expresses high levels of the estrogen and progesterone receptors (PR) and is rarely associated with overexpression or amplification of human epidermal growth factor receptor 2 (HER2) [10, 16]. However, pleomorphic lobular carcinoma (PLC) has been known to lose expression of the hormone receptors and can be associated with HER2 amplification. Therefore, PLC is more aggressive, reflected by the larger tumor size, higher-grade cytological features, enhanced Ki-67 expression rates, higher incidence of distant metastasis (DM), presence of lymphovascular invasion (LVI), more advanced stage at presentation, and poor clinical outcome [17, 18]. Information on the biological structures, clinical behaviors, and effective treatment strategies for ILC variants other than PLC is limited [19].

An understanding of the differences among cILC, PLC, and the other ILC variants is crucial for accurate diagnosis, risk stratification, and tailored treatment planning. This article compares variants of ILC according to their clinical outcomes and histopathological features.

## Patients and methods

Patients diagnosed with ILC and treated at the University of Health Sciences, Turkiye, Istanbul Haydarpasa Numune Training and Research Hospital between January 2010 and August 2021 were retrospectively evaluated. Ethics approval for the study was obtained from the institutional ethics committee (approval no. HNEAH-KAEK 2024/KK/27).

Patients with isolated lobular carcinoma in situ, invasive ductal carcinoma or inflammatory breast cancer, bilateral breast cancer, distant metastasis at diagnosis, a lack of clinical follow-up data, no definitive surgical treatment, or no data on histopathological features, and treated with neoadjuvant chemotherapy were excluded.

Patients were divided into three groups according to their histopathological features; group 1: cILC; group 2: PLC; group 3: mixed ILC. Mixed ILC was divided into three subgroups: 3a, cILC + PLC; 3b, cILC + mixed; 3c, PLC + mixed (Table 1).

**Table 1:** Histopathological classification and clinicopathological features of the groups

	n	%		
Total	254	100		
Group 1 (cILC)	136	53.5		
Group 2 (PLC)	27	10.6		
Group 3 (Mixed ILC)	91	35.8		n
Group 3a (cILC + PLC)	46	18.1	cILC + PLC	46
Group 3b (cILC + mixed)	19	7.5	cILC + aILC + tlILC	1
			cILC + tlILC	12
			cILC + srILC	6
Group 3c (PLC + mixed)	26	10.2	PLC + mILC	1
			PLC + hILC	1
			PLC + aILC	3
			PLC + tlILC	2
			PLC + srILC	13
			PLC + cILC + hILC	2
			PLC + cILC + srILC	4

	Minimum–maximum (Median)	Mean (SD)	
Age (years)	30–90 (57)	58.3 (SD 11.9)	
	≤ 49 years	69	27.2
	50–59 years	75	29.5
	60–69 years	64	25.2
	≥ 70 years	46	18.1
Tumor size (millimeter)	2–150 (31.5)	39.1 (SD 26.1)	
Ki-67 (%)	2–90 (15)	20.1 (SD 17.4)	
Disease free survival (month)	8–156 (46)	53.9 (SD 34.2)	
Overall survival (month)	12–156 (48)	56.7 (SD 33.6)	

Molecular subtype	LA	149	58.7
	LB. HER2-negative	90	35.4
	LB. HER2-positive	7	2.8
	Triple negative	8	3.1
Nuclear grade	Grade I	53	20.9
	Grade II	147	57.9
	Grade III	54	21.3
Lymphovascular invasion	No	182	71.7
	Yes	72	28.3
Multifocality	No	133	52.4
	Yes	121	47.6
pN	pN0	122	48.0
	pN1a	72	28.3
	pN2a	31	12.2
	pN3a	29	11.4
Axillary surgery	SLNB	144	56.7
	ALND	110	43.3
Breast surgery	BCS	111	43.7
	Mastectomy	143	56.3
Locoregional recurrence	No	239	94.1
	Yes	15	5.9
Distant metastasis	No	231	90.9
	Yes	23	9.1
Mortality	No	238	93.7
	Yes	16	6.3

***n*** number, ***cILC*** classic invasive lobular carcinoma, ***PLC*** pleomorphic lobular carcinoma, ***aILC*** alveolar invasive lobular carcinoma, ***tlILC*** tubulolobular invasive lobular carcinoma, ***srILC*** signet-ring cellinvasive lobular carcinoma, ***mILC*** mucinous invasive lobular carcinoma, ***hILC*** histiocytoid invasive lobular carcinoma, ***SD*** standard deviation, ***LA*** Luminal A, ***LB*** Luminal B, ***HER2*** human epidermal growth factor receptor 2, ***pN*** pathological axillary lymph node, ***SLNB*** sentinel lymph node biopsy, ***ALND*** axillary lymph node dissection, ***BCS*** breast conserving surgery

Clinicopathological variables such as age, tumor size, nuclear grade, LVI status, tumor multifocality, pathological lymph node stage (pN), and type of surgical treatment were used to evaluate the groups. Data were obtained from pathology reports.

pN was divided into four subgroups: pN0, reactive lymph nodes; and pN1–3, 1–3, 4–9, and ≥ 10 metastatic lymph nodes, respectively.

Breast surgery was divided into two types: breast-conserving surgery (BCS) and mastectomy. Axillary surgery was also divided into two types: sentinel lymph node biopsy (SLNB) and axillary lymph node dissection (ALND).

In terms of ER and PR status, tumors with nuclear expression levels ≥ 1% were considered positive. In terms of HER2, patients with immunohistochemical (IHC) scores of 3 + or 2 + , and who exhibited gene amplification on fluorescence in situ hybridization, were considered HER2-positive. A Ki-67 threshold value ≥ 20% was used to distinguish luminal A (LA) and luminal B (LB) breast cancer. Based on the IHC results for the hormone receptors, HER2, and Ki-67, tumors were classified into five molecular subtypes: LA (ER-positive and/or PR-positive, HER2-negative, and Ki-67 < 20%); LB, HER2-negative (ER-positive and/or PR-positive, Ki-67 ≥ 20%, HER2-negative); LB, HER2-positive (ER-positive and/or PR-positive, HER2-positive); HER2-positive (ER/PR-negative, HER2-positive); and triple-negative (TN; ER/PR/HER2-negative). No patient had the HER2-positive subtype.

Additionally, the following event-related data were collected: locoregional recurrence (LRR) or DM, disease-free survival (DFS), overall survival (OS), and cancer-related mortality. OS was defined as the period from surgery to death from any cause or the last follow-up. DFS was defined as the interval between surgery and LRR, DM, or death from breast cancer, whichever occurred first.

## Statistical analyses

IBM SPSS Statistics 26 (IBM SPSS, Turkiye) software was used for the statistical analysis. The study data are presented as descriptive statistics such as the mean, standard deviation, median, frequency, ratio, minimum, and maximum. The normality of the distribution of the quantitative data was evaluated graphically and using the Kolmogorov–Smirnov, Shapiro–Wilk, and skewness-kurtosis tests.

One-way ANOVA was used to compare three or more groups with normally distributed variables, and the Bonferroni test was employed for pairwise comparisons. The Kruskal–Wallis test was used to compare three or more groups for which the variables were not normally distributed, and the Bonferroni-Dunn test was employed for pairwise comparisons. The Pearson chi-square test and Fisher-Freeman-Halton exact test were used to compare qualitative data. Kaplan–Meier survival analysis was also performed, and the log-rank test was used to evaluate survival. The significance level was set to *p* < 0.05.

## Results

The age of the 254 women included in our study ranged from 30 to 90 years (median age, 57 years). Patients were evaluated after grouping them into histopathological subgroups (Table 1): Group 1 (cILC), 136 patients; Group 2 (PLC), 27 patients; Group 3 (Mixed ILC), 91 patients; Group 3a (cILC + PLC), 46 patients; Group 3b (cILC + mixed), 19 patients; and Group 3c (PLC + mixed), 26 patients. Except for pure cILC and PLC, no other pure variant ILCs were found.

Median overall survival (OS) at follow-up was 48 months, and median disease-free survival (DFS) was 46 months. Locoregional recurrence (LRR) occurred in 15 (5.9%) of the patients, and distant metastasis (DM) developed in 23 (9.1%). Breast cancer-related death occurred in 16 (6.3%) patients (Tablo 1).

The median tumor size was smaller in Group 1 (32.7 mm) than in Group 2 and 3 (44.7 mm and 47.1 mm, respectively). The median Ki-67 value was also lower in Group 1 (Table 2). In Groups 1 and 3b, where the PLC variant was not present, the rates of nuclear grade III were 8.1% and 0%, respectively. In contrast, in Groups 2, 3a, and 3c, where the PLC variant was present, the rates of nuclear grade III increased to 40.7%, 39.1%, and 53.8%, respectively (Table 3). The incidence of aggressive molecular subtypes (LB, HER2-positive, TN) was statistically higher in Group 2. Only 0.7% of patients were LB, HER2-positive in Group 1, whereas this rate was 7.4% in Group 2. In Groups 1, 2, and 3, 33.8%, 59.3%, and 64.8% of patients were multifocal, respectively. Pleomorphic and mixed variants were approximately twice as likely to be multifocal as the classic variant. No significant differences were found between the groups in terms of pN stage and type of axillary surgery. Mastectomy was performed in 41.9% of the patients in Group 1, compared to 77.8% and 71.4% in Groups 2 and 3, respectively. No statistically significant differences were observed between the groups regarding LRR rates. The incidence of DM was 4.4% in Group 1, 22.2% in Group 2 and 12.1% in Group 3. Mortality rates were 3.7%, 18.5%, and 6.6% in Groups 1, 2, and 3, respectively.

**Table 2:** Evaluation of the clinicopathological features of patients in the three subgroups

		Histopathology			p
		cILC (n:136) Group 1	PLC (n:27) Group 2	Mixed ILC (n:91) Group 3	
		Min–max (Median) Mean (SD)	Min–max (Median)Mean (SD)	Min–max (Median)Mean (SD)	
Age (years)		35–90 (57) 58.8 (SD 12.0)	38–85 (63) 63.7 (SD 13.9)	30–82 (55) 55.9 (SD 10.7)	^a^0.009**
	≤ 49 years	32 (23.5)	5 (18.5)	32 (35.2)	^c^0.093
	50–59 years	44 (32.4)	6 (22.2)	25 (27.5)	
	60–69 years	33 (24.3)	7 (25.9)	24 (26.4)	
	≥ 70 years	27 (19.9)	9 (33.3)	10 (11.0)	
Tumor size (millimeter)		2–125 (28) 32.7 (SD 22.0)	10–150 (35) 44.4 (SD 31.0)	10–145 (40) 47.1 (SD 27.9)	^b^0.001**
Ki-67 (%)		2–70 (10) 15.6 (SD 12.5)	5–70 (26) 27 (SD 15.8)	2–90 (20) 24.9 (SD 21.9)	^b^0.001**
Disease free survival (month)		10–156 (54.5) 60.3 (SD 34.6)	12–120 (43) 46.6 (SD 28.6)	8–140 (37) 46.3 (SD 33.6)	^b^0.001**
Overall survival (month)		12–156 (57) 62.5 (SD 33.3)	15–120 (48) 52 (SD 28.1)	15–140 (38) 49.4 (SD 34.2)	^b^0.001**

		n (%)	n (%)	n (%)	p
Molecular subtype	LA	95 (69.9)	10 (37)	44 (48.4)	^d^0.001**
	LB. HER2-negative	37 (27.2)	12 (44.4)	41 (45.1)	
	LB. HER2-positive	1 (0.7)	2 (7.4)	4 (4.4)	
	Triple negative	3 (2.2)	3 (11.1)	2 (2.2)	
Nuclear grade	Grade I	44 (32.4)	1 (3.7)	8 (8.8)	^c^0.001**
	Grade II	81 (59.6)	15 (55.6)	51 (56)	
	Grade III	11 (8.1)	11 (40.7)	32 (35.2)	
Lymphovascular invasion	No	107 (78.7)	20 (74.1)	55 (60.4)	^c^0.011*
	Yes	29 (21.3)	7 (25.9)	36 (39.6)	
Multifocality	No	90 (66.2)	11 (40.7)	32 (35.2)	^c^0.001**
	Yes	46 (33.8)	16 (59.3)	59 (64.8)	
pN	pN0	71 (52.2)	12 (44.4)	39 (42.9)	^c^0.365
	pN1a	39 (28.7)	5 (18.5)	28 (30.8)	
	pN2a	14 (10.3)	6 (22.2)	11 (12.1)	
	pN3a	12 (8.8)	4 (14.8)	13 (14.3)	
Axillary surgery	SLNB	85 (62.5)	15 (55.6)	44 (48.4)	^c^0.107
	ALND	51 (37.5)	12 (44.4)	47 (51.6)	
Breast surgery	BCS	79 (58.1)	6 (22.2)	26 (28.6)	^c^0.001**
	Mastectomy	57 (41.9)	21 (77.8)	65(71.4)	
Locoregional recurrence	No	128 (94.1)	26 (96.3)	85 (93.4)	^c^0.855
	Yes	8 (5.9)	1 (3.7)	6 (6.6)	
Distant metastasis	No	130 (95.6)	21 (77.8)	80 (87.9)	^c^0.006**
	Yes	6 (4.4)	6 (22.2)	11 (12.1)	
Mortality	No	131 (96.3)	22 (81.5)	85 (93.4)	^c^0.015*

^a^One-way ANOVA Test, ^b^Kruskal Wallis Test, ^c^Pearson Chi-Square Test, ^d^Fisher Freeman Halton Exact Test, *p < 0.05, **p < 0.01

***cILC*** classic invasive lobular carcinoma,*** n*** number, ***PLC*** pleomorphic lobular carcinoma, ***min*** minimum, ***max*** maximum, ***SD*** standard deviation, ***LA*** Luminal A, ***LB*** Luminal B, ***HER2*** human epidermal growth factor receptor 2, ***pN*** pathological axillary lymph node, ***SLNB*** sentinel lymph node biopsy, ***ALND*** axillary lymph node dissection, ***BCS*** breast conserving surgery

**Table 3:** Evaluation of the clinicopathological features of patients in the five subgroups

		Histopathology					p
		cILC (n:136) Group 1	PLC (n:27) Group 2	cILC + PLC (n:46) Group 3a	cILC + mixed (n:19) Group 3b	PLC + mixed (n: 26) Group 3c	
		Min–max (Median) Mean (SD)	Min–max (Median) Mean (SD)	Min–max (Median) Mean (SD)	Min–max (Median) Mean (SD)	Min–max (Median) Mean (SD)	
Age (years)		35–90 (57) 58.8 (SD 12.0)	38–85 (63) 63.7 (SD 13.9)	35–82 (55) 56.1 (SD 10.8)	42–70 (57) 55.8 (SD 7.7)	30–82 (54) 55.6 (SD 12.7)	^a^0.083
	≤ 49 years	32 (23.5)	5 (18.5)	15 (32.6)	5 (26.3)	12 (46.2)	^d^0.072
	50–59 years	44 (32.4)	6 (22.2)	15 (32.6)	8 (42.1)	2 (7.7)	
	60–69 years	33 (24.3)	7 (25.9)	10 (21.7)	5 (26.3)	9 (34.6)	
	≥ 70 years	27 (19.9)	9 (33.3)	6 (13)	1 (5.3)	3 (11.5)	
Tumor size (millimeter)		2–125 (28) 32.7 (SD 22.0)	10–150 (35) 44.4 (SD 31.0)	12–125 (50) 50.2 (SD 29.7)	22–145 (39) 50.4 (SD 30.8)	10–85 (35) 39.1 (SD 20.9)	^b^0.001**
Ki-67 (%)		2–70 (10) 15.6 (SD 12.5)	5–70 (26) 27 (SD 15.8)	5–90 (15) 22.7 (SD 22.1)	2–40 (10) 14.1 (SD 10.6)	5–80 (30) 36.8 (SD 22.8)	^b^0.001**
Disease free survival (month)		10–156 (54.5) 60.3 (SD 34.6)	12–120 (43) 46.6 (SD 28.6)	12–140 (36) 46.7 (SD 36.4)	8–115 (42) 54.5 (SD 34.4)	10–120 (30.5) 39.6 (SD 27.0)	^b^0.002**
Overall survival (month)		12–156 (57) 62.5 (SD 33.3)	15–120 (48) 52 (SD 28.1)	15–140 (39.5) 50.8 (SD 38.1)	18–115 (42) 56.7 (SD 33.7)	15–120 (35) 41.5 (SD 25.8)	^b^0.003**

	n (%)	n (%)	n (%)	n (%)	n (%)	p	
Molecular subtype	LA	95 (69.9)	10 (37)	28 (60.9)	11 (57.9)	5 (19.2)	^d^0.001**
	LB. HER2-negative	37 (27.2)	12 (44.4)	16 (34.8)	8 (42.1)	17 (65.4)	
	LB. HER2-positive	1 (0.7)	2 (7.4)	1 (2.2)	0 (0)	3 (11.5)	
	Triple negative	3 (2.2)	3 (11.1)	1 (2.2)	0 (0)	1 (3.8)	
Nuclear grade	Grade I	44 (32.4)	1 (3.7)	2 (4.3)	6 (31.6)	0 (0)	^c^0.001**
	Grade II	81 (59.6)	15 (55.6)	26 (56.5)	13 (68.4)	12 (46.2)	
	Grade III	11 (8.1)	11 (40.7)	18 (39.1)	0 (0)	14 (53.8)	
Lymphovascular invasion	No	107 (78.7)	20 (74.1)	27 (58.7)	14 (73.7)	14 (53.8)	^c^0.024*
	Yes	29 (21.3)	7 (25.9)	19 (41.3)	5 (26.3)	12 (46.2)	
Multifocality	No	90 (66.2)	11 (40.7)	16 (34.8)	10 (52.6)	6 (23.1)	^c^0.001**
	Yes	46 (33.8)	16 (59.3)	30 (65.2)	9 (47.4)	20 (76.9)	
pN	pN0	71 (52.2)	12 (44.4)	19 (41.3)	6 (31.6)	14 (53.8)	^d^0.553
	pN1a	39 (28.7)	5 (18.5)	13 (28.3)	8 (42.1)	7 (26.9)	
	pN2a	14 (10.3)	6 (22.2)	6 (13)	3 (15.8)	2 (7.7)	
	pN3a	12 (8.8)	4 (14.8)	8 (17.4)	2 (10.5)	3 (11.5)	
Axillary surgery	SLNB	85 (62.5)	15 (55.6)	22 (47.8)	6 (31.6)	16 (61.5)	^c^0.075
	ALND	51 (37.5)	12 (44.4)	24 (52.2)	13 (68.4)	10 (38.5)	
Breast surgery	BCS	79 (58.1)	6 (22.2)	15 (32.6)	4 (21.1)	7 (26.9)	^d^0.001**
	Mastectomy	57 (41.9)	21 (77.8)	31 (67.4)	15 (78.9)	19 (73.1)	
Locoregional recurrence	No	128 (94.1)	26 (96.3)	43 (93.5)	18 (94.7)	24 (92.3)	^d^0.972
	Yes	8 (5.9)	1 (3.7)	3 (6.5)	1 (5.3)	2 (7.7)	
Distant metastasis	No	130 (95.6)	21 (77.8)	40 (87)	17 (89.5)	23 (88.5)	^d^0.019*
	Yes	6 (4.4)	6 (22.2)	6 (13)	2 (10.5)	3 (11.5)	
Mortality	No	131 (96.3)	22 (81.5)	42 (91.3)	19 (100)	24 (92.3)	^d^0.042*
	Yes	5 (3.7)	5 (18.5)	4 (8.7)	0 (0)	2 (7.7)	

^a^One-way ANOVA Test, ^b^Kruskal Wallis Test, ^c^Pearson Chi-Square Test, ^d^Fisher Freeman Halton Exact Test, *p < 0.05, **p < 0.01

***cILC*** classic invasive lobular carcinoma, ***PLC*** pleomorphic lobular carcinoma, ***min*** minimum, ***max*** maximum, ***SD*** standard deviation, ***n*** number, ***LA*** Luminal A, ***LB*** Luminal B, ***HER2*** human epidermal growth factor receptor 2, ***pN*** pathological axillary lymph node, ***SLNB*** sentinel lymph node biopsy, ***ALND*** axillary lymph node dissection, ***BCS*** breast conserving surgery

Among the patients who underwent BCS, a second operation was planned for 27 (23%) patients due to positive surgical margins. Among these patients, 6 were converted from BCS to mastectomy. In 21 patients, surgical margins were re-excised with BCS, and surgical margin negativity was achieved. Of the 27 patients, 18 were in Group 1, 2 were in Group 2, and 7 were in Group 3. In total, out of 254 patients, BCS was performed in 111, and mastectomy was performed in 143. Of the 15 (5.9%) patients who developed LRR, BCS was performed in 10, and mastectomy was performed in 5. Of the patients who developed LRR, eight were in Group 1, one was in Group 2, and six were in Group 3. There was no significant difference in LRR rate among groups.

**Fig. 1:**
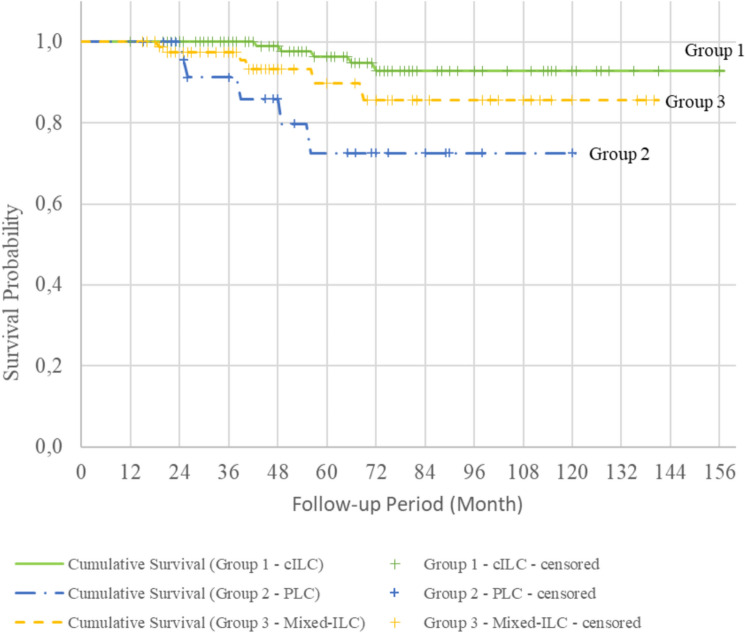
Kaplan–Meier survival curves of overall survival according to the three subgroups. *cILC* classic invasive lobular carcinoma, *PLC* pleomorphic lobular carcinoma, *ILC* invasive lobular carcinoma

**Fig. 2:**
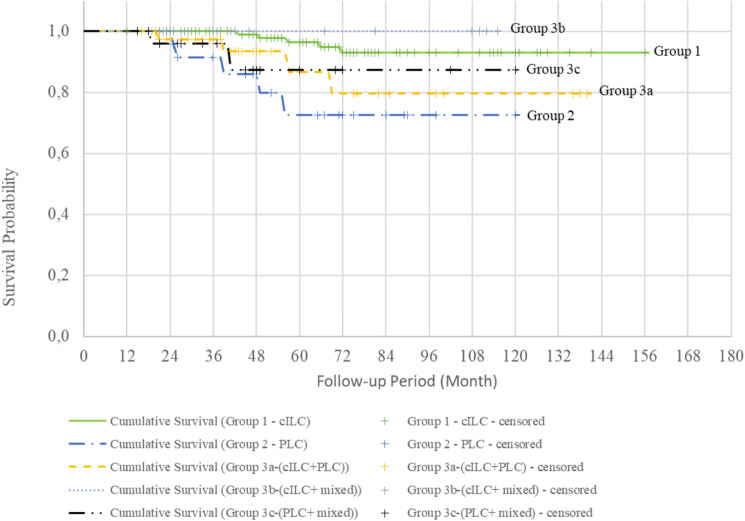
Kaplan–Meier survival curves of overall survival according to the five subgroups. *cILC* classic invasive lobular carcinoma, *PLC* pleomorphic lobular carcinoma

**Fig. 3:**
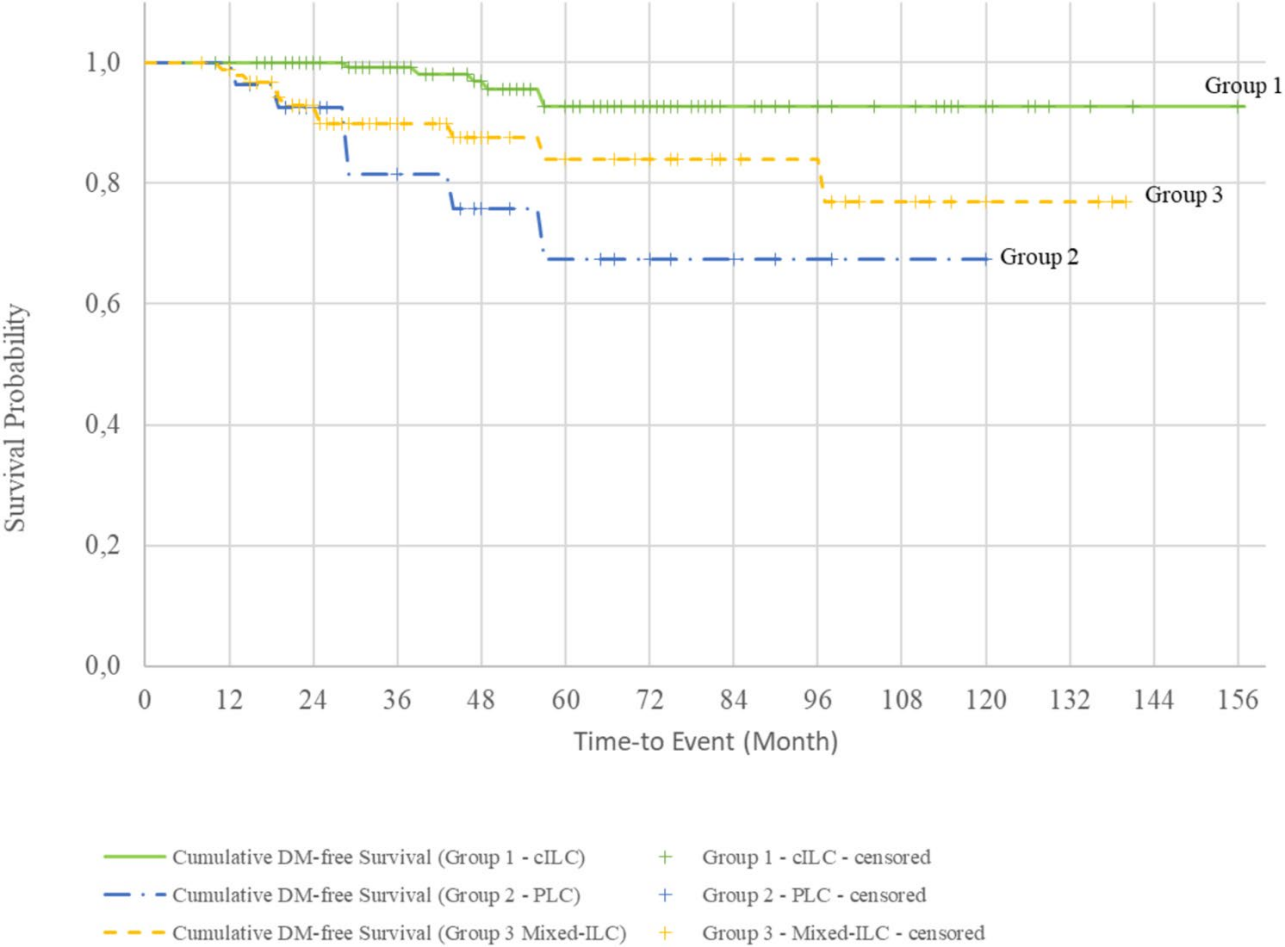
Kaplan–Meier survival curves of distant metastasis (DM)-free survival according to the three subgroups. *cILC* classic invasive lobular carcinoma, *PLC* pleomorphic lobular carcinoma, *ILC* invasive lobular carcinoma

**Fig. 4:**
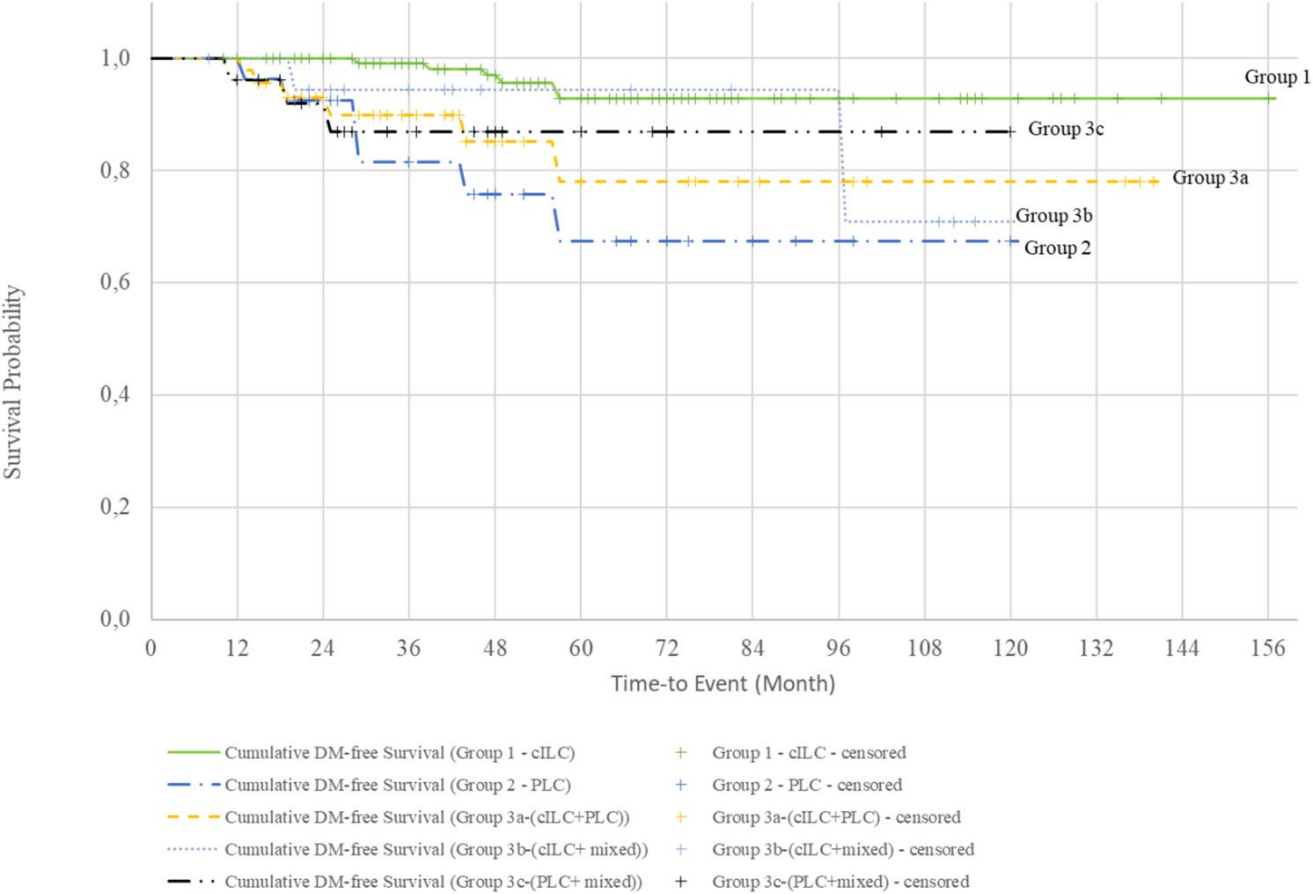
Kaplan–Meier survival curves of distant metastasis (DM)-free survival according to the five subgroups. *cILC* classic invasive lobular carcinoma, *PLC* pleomorphic lobular carcinoma

**Fig. 5:**
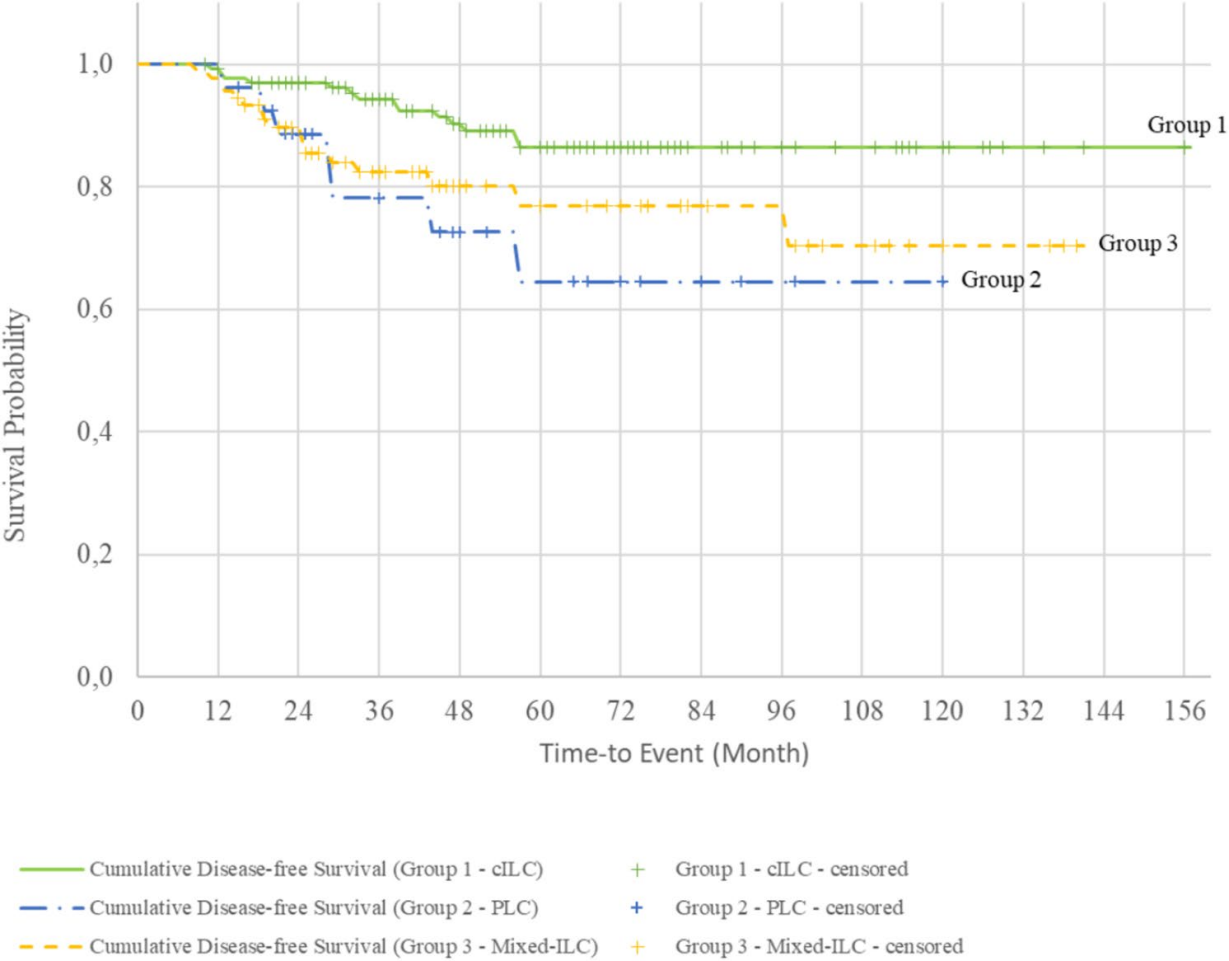
Kaplan–Meier survival curves of disease-free survival (DFS) according to the three subgroups. *cILC* classic invasive lobular carcinoma, *PLC* pleomorphic lobular carcinoma, *ILC* invasive lobular carcinoma

The cumulative survival rates for Groups 1, 2, and 3 were 92.9%, 72.5%, and 85.6%, respectively (Fig. 1). Survival curves evaluated with the log-rank test showed that patients with the PLC variant had a significantly lower survival rate than those with pure cILC (Fig. 2, Table 4). The cumulative DM-free survival rates were 92.8%, 67.3%, and 76.9% in Groups 1, 2, and 3, respectively. Evaluation of the survival curves with the log-rank test revealed a statistically lower DM-free survival rate for all groups containing the PLC variant compared with that of patients with pure cILC (Figs. 3 and 4). Additionally, the DFS of Group 2 and 3 patients was lower than that of Group 1 patients (Fig. 5).

**Table 4:** Survival analysis of the subgroups

	n	Locoregional recurrence (LRR)		LRR-free survival %	Cumulative LRR-free survival	95% Confidence Interval		Log Rank Test; p
		Yes	No			Lower	Upper	
Group 1	136	8	128	94.1	147.12 (SD 3.04)	141.152	153.081	0.785
Group 2	27	1	26	96.3	115.83 (SD 4.08)	107.839	123.828	
Group 3	91	6	85	93.4	130.08 (SD 3.93)	122.367	137.785	
Group 1	136	8	128	94.1	147.12 (SD 3.04)	141.152	153.081	0.950
Group 2	27	1	26	96.3	115.83 (SD 4.08)	107.839	123.828	
Group 3a	46	3	43	93.5	129.45 (SD 5.88)	117.933	140.979	
Group 3b	19	1	18	94.7	109.37 (SD 5.48)	98.625	120.112	
Group 3c	26	2	24	92.3	109.37 (SD 7.26)	95.135	123.608	

	n	Distant Metastasis (DM)		DM-free survival %	Cumulative DM-free survival	95% Confidence Interval		Log Rank Test; p
		Yes	No			Lower	Upper	
Group 1	136	6	130	95.6	148.14 (SD 3.11)	142.048	154.237	0.001**
Group 2	27	6	21	77.8	92.19 (SD 9.64)	73.303	111.083	
Group 3	91	11	80	87.9	119.29 (SD 5.99)	107.544	131.034	
Group 1	136	6	130	95.6	148.14 (SD 3.11)	142.048	154.237	0.008**
Group 2	27	6	21	77.8	92.19 (SD 9.64)	73.303	111.083	
Group 3a	46	6	40	87.0	117.09 (SD 8.85)	99.737	134.443	
Group 3b	19	2	17	89.5	105.18 (SD 6.27)	92.883	117.478	
Group 3c	26	3	23	88.5	106.60 (SD 7.26)	92.371	120.829	

	n	Recurrence (LRR + DM)		Disease free survival (DFS) %	Cumulative DFS	95% Confidence Interval		Log Rank Test; p
		Yes	No			Lower	Upper	
Group 1	136	14	122	89.7	139.78 (SD 4.07)	131.797	147.766	0.014*
Group 2	27	7	20	74.1	89.17 (SD 9.70)	70.151	108.188	
Group 3	91	17	74	81.3	110.99 (SD 6.40)	98.438	123.542	
Group 1	136	14	122	89.7	139.78 (SD 4.07)	131.797	147.766	0.059
Group 2	27	7	20	74.1	89.17 (SD 9.70)	70.151	108.188	
Group 3a	46	9	37	80.4	108.47 (SD 9.38)	90.089	126.848	
Group 3b	19	3	16	84.2	100.07 (SD 7.75)	84.869	115.262	
Group 3c	26	5	21	80.8	97.33 (SD 9.09)	79.524	115.142	

	n	Mortality		Overall survival (OS) %	Cumulative survival	95% Confidence Interval		Log Rank Test; p
		Yes	No			Lower	Upper	
Group 1	136	5	131	96.3	149.07 (SD 3.01)	143.163	154.977	0.007**
Group 2	27	5	22	81.5	98.16 (SD 8.47)	81.550	114.764	
Group 3	91	6	85	93.4	126.80 (SD 5.25)	116.517	137.087	
Group 1	136	5	131	96.3	149.07 (SD 3.01)	143.163	154.977	0.013*
Group 2	27	5	22	81.5	98.16 (SD 8.47)	81.550	114.764	
Group 3a	46	4	42	91.3	121.98 (SD 8.35)	105.619	138.339	
Group 3b	19	0	19	100	107.33 (SD 7.23)	93.166	121.501	
Group 3c	26	2	24	92.3	108.94 (SD 7.62)	93.999	123.878	

Kaplan Meier Survival analysis and Log Rank test*p<0.05, **p<0.01

***n*** number, ***LRR*** Locoregional recurrence, ***SD*** standard deviation, ***DM ***Distant Metastasis, ***DFS*** Disease free survival, ***OS*** Overall survival

## Discussion

ILC exhibits specific behavioral patterns, and each variant has distinct characteristics. Among them, PLC is associated with the poorest outcomes and prognostic factors [12, 20, 21]. However, the prognostic significance of other variants has not yet been fully elucidated, and prognosis depends on multiple factors, including molecular behavior, tumor size, grade, and stage [10, 19].

ILC is more commonly seen in older individuals, and advanced age is associated with worse clinical outcomes [7, 20]. Our data, along with previous studies [18, 22] also showed that patients with PLC tend to be older.

Compared to cILC, PLC is often strongly associated with poor prognostic factors such as higher nuclear grade, higher Ki-67 expression, lower expression of hormone receptors (HR), and high rates of HER2 expression [6, 7, 15, 20, 23, 24]. On the contrary, Bozkurt et al. did not find any significant difference in ER/PR/HER2 status among variants in their study [19]. In our study, all groups containing the pleomorphic variant had a higher nuclear grade than the cILC group. Although Ki-67 expression percentages were highest in the PLC and PLC-mixed groups, Ki-67 expression decreased when tumors contained the classic variant. In the PLC group, patients were significantly more likely to be triple negative and luminal B HER2 positive (LB, HER2 +) than the cILC group. As the tumor contained variants other than the classic variant, we found that the molecular subtype shifted to a more aggressive subtype.

Most studies have shown that PLCs are usually large at the time of diagnosis and are often multifocal [18, 22, 25]. When we analyzed the mean tumor size and multifocality rates, we observed that the cILC subgroup had the smallest tumor size; Group 3b had a larger tumor size than Group 1, even though it did not contain the pleomorphic variant. Also, the groups that contained pleomorphic variants had the highest multifocality rates.

ILC, despite favorable prognostic features such as positive hormone receptor status, HER2-negativity, low nuclear grade, and a low to moderate Ki-67 proliferation index, is generally diagnosed at a more advanced stage and is associated with a higher incidence of axillary lymph node (ALN) involvement compared with IDC [25–27]. Although numerous studies have investigated ALN involvement in ILC and IDC, studies specifically comparing ALN status among the various ILC variants remain limited. In our study, 51.9% of all ILC patients had ALN metastases. The PLC groups in previous studies had higher rates of ALN metastasis than the cILC groups [18, 28, 29]. However, in the study by Buchanan et al., the data set included only patients with clinically negative axilla [29]. In contrast, several published studies argue against these findings [18]. Narendra et al. stated that 51% of PLC patients had ALN metastases, and Jacob et al. found no statistically significant difference in LVI or ALN status between PLC and cILC groups [22, 23]. Likewise, in our study, there was no statistically significant difference in ALN status among the groups. However, mixed groups with pleomorphic variants had significantly higher LVI rates than the cILC group. The lack of difference in ALN status may be explained by the fact that patients with pleomorphic variants and positive ALNs were more commonly referred for neoadjuvant systemic therapy (NST), and those who received this treatment were excluded from the study.

The clinical management and surgical treatment of ILC remains controversial. ILC is a distinct subtype of breast cancer that deserves special attention by surgeons [3]. Achieving clear surgical margins is also a major challenge. Mastectomy rates remain high, and patients undergoing BCS are more likely to have positive resection margins. According to the literature, re-excision rates among ILC patients undergoing BCS range from 17 to 65% [2, 7, 10, 17, 18]. In our study, the mastectomy rate was 56.3% after considering many factors, including the larger tumor size at the time of diagnosis, location (more multifocal or multicentric), and the tendency to grow diffusely (thus requiring re-excision more often to clear the margin) [3, 4, 10, 14]. However, well-localized and small tumors without clinical or radiologic evidence of multifocality or multicentricity can be treated with BCS [17]. In our cohort, BCS rates were higher in the cILC group than in the other groups, and no significant difference was found in the LRR rate among the groups. Among the patients who underwent BCS, 23% required re-excision due to positive surgical margins, and only six patients underwent completion mastectomy. The previous study reported that, among ILC patients who underwent completion mastectomy, 24% had benign mastectomy specimens. Additionally, the median size of the remaining tumor among those with residual disease was only 0.6 cm, suggesting that achieving negative margins with re-excision lumpectomy is possible [10]. In the study by Sagdıc et al., the completion mastectomy rate was higher in the PLC group [30]. In our study, mastectomy was performed in 41.9% of the patients in Group 1, compared to 77.8% and 71.4% in Groups 2 and 3, respectively. Among patients who underwent re-excision, only two were from Group 2. Mastectomy rates were nearly twice as high in the PLC group compared to the cILC group, likely reflecting our surgical team’s concerns about achieving clear surgical margins, as also noted by Sagdıc et al. Recent studies have found that BCS and mastectomy do not differ in the local control of ILC [10, 31]. These findings support the high feasibility of BCS, provided that clear surgical margins are achieved [32, 33]. Peradze et al. also reported that the surgical approach did not significantly influence OS and DFS in patients with ILC, and although they identified negative prognostic factors in the pleomorphic subtype when comparing PLC with cILC, the therapeutic approach and management of both histotypes have remained similar [34].

ILC has a higher risk of late DM and may exhibit more aggressive metastatic behavior, resulting in worse long-term outcomes compared to IDC at the same stage [35]. Makhlouf et al. examined an aggressive ILC subtype that includes PLC and high-grade solid ILC. These tumors were associated with clinicopathological features of poor prognosis and had higher BC-specific mortality and recurrence rates compared to both cILC and IDC patients [11]. Similarly, Liu et al. noted that early studies reported worse OS and DFS rates, as well as higher recurrence rates, in PLC compared to ILC [36]; however, some studies found similar clinical outcomes [18, 22, 23, 36]. In this study, although there was no significant difference in LRR rate among all subgroups, we found that mortality and DM rates were higher, in the PLC and mixed ILC groups. In the analysis of three groups (Groups 1, 2, and 3), the median OS was 62.5, 52.0, and 49.4 months, and the median DFS was 60.3, 46.6, and 46.3 months, respectively. When the analysis was extended to five groups (Groups 1, 2, 3a, 3b, and 3c), the median OS was 62.5, 52.0, 50.8, 56.7, and 41.5 months, while the median DFS was 60.3, 46.6, 46.7, 54.5, and 39.6 months, respectively. Notably, the PLC + mixed group without a classic variant (Group 3c) demonstrated even poorer outcomes than pure PLC.

It is important to acknowledge the limitations of our study. Its retrospective design and relatively small sample size may have introduced selection bias, potentially limiting the generalizability of our findings. Furthermore, the study design did not allow us to fully explore the rationale behind surgical treatment choices. To ensure standardization, we did not include patients who received NST in our study. However, in ILC patients, the low response rates to NST, particularly in clinical ALN positive patients, may have influenced treatment decisions, and affected the assessment of ALN status. As a result, this may have affected the characteristics of the patients included in the study.

## Conclusion

Although numerous studies in the literature have compared ILC, PLC, and IDC, this study is one of the few that comprehensively evaluates all histological variants of ILC. In our analysis, pure cILC exhibited the best prognostic features among the ILC subtypes. While pure PLCs showed distinctly aggressive pathological characteristics, we identified that PLC combined with non-classic variants was associated with the poorest survival outcomes. Furthermore, we observed a higher mastectomy rate in patients with pleomorphic variants, which might possibly—though not conclusively—explain the absence of a statistically significant difference in LRR among the variants. Nevertheless, since aggressive prognostic features remain the key determinants of survival, we would also like to emphasize that oncoplastic BCS may be considered when negative surgical margins can be achieved. Moreover, comprehensive randomized controlled trials are essential to establish standardized treatment protocols for ILC patients. Additionally, there is an increasing need for the development and exploration of innovative therapeutic modalities to enhance patient outcomes.

## Data Availability

The data supporting the findings of this study are available from the corresponding author upon reasonable request.
